# Sleeping Sickness

**Published:** 1919-05

**Authors:** 


### Sleeping Sickness.

The best medical talent of the country is now engaged in a study of lethargic encephalitis, which is slowly spreading over the entire country. The symptoms are not always the same, but the diagnosis is in practically every case correct, although a few of the reported cases have proven to be something else. The health departments of Texas and adjoining states are making close investigations of the cases reported, together with the treatments that appear to be most effective. The Texas Medical Journal is in close touch with these departments and has the promise of early reports of the results of the investigations and will give as much information on the subject as can be obtained. Several reports will be found in this number and others will be published in June and in succeeding issues. Doctors everywhere are interested and are anxious to learn everything possible about this insidious disease, which is generally regarded as the aftermath of the epidemic of influenza.

